# Performance metrics and enabling technologies for nanoplasmonic biosensors

**DOI:** 10.1038/s41467-018-06419-3

**Published:** 2018-12-10

**Authors:** Sang-Hyun Oh, Hatice Altug

**Affiliations:** 1 Department of Electrical and Computer Engineering, University of Minnesota, Minneapolis, MN 55455 USA; 2 Institute of Bioengineering, École Polytechnique Fédérale de Lausanne (EPFL), Lausanne, 1015 Switzerland

## Abstract

Nanoplasmonic structures can tightly confine light onto a material’s surface to probe biomolecular interactions not easily accessed by other sensing techniques. New and exciting developments in nanofabrication processes, nano-optical trapping, graphene devices, mid-infrared spectroscopy, and metasurfaces will greatly empower the performance and functionalities of nanoplasmonic sensors.

## Introduction

The emerging field of nanoplasmonics has profoundly impacted the development of bioanalytical sensors^1^. Following the commercial success of surface plasmon resonance (SPR) biosensors in pharmaceutical R&D, researchers have strived to improve their performance by replacing thin gold films in SPR sensors with engineered “building blocks” of nanoplasmonics such as nanometer-scale holes, particles, and gaps. Advances in nanofabrication have enabled researchers to produce these metallic nanostructures with high throughput and resolution^2^. However, to justify the added cost and complexity of nanofabrication, we think that these engineered nanoplasmonic devices should extend beyond merely improving the sensitivity of the device and offer new functionalities that conventional SPR sensors cannot. We share our views on promising directions for nanoplasmonic sensors along with new and exciting challenges awaiting researchers.

## Conventional performance metrics in plasmonic sensing

We begin with commonly used performance metrics for evaluating nanoplasmonic sensors: bulk sensitivity (resonance shifts per bulk refractive index (RI) change), thin-film sensitivity (resonance shifts per added film thickness), and the figure-of-merit (FOM): (resonance shifts)/(linewidth). These metrics alone, however, are not sufficient to describe the detection limit of sensors. It is important to also consider the parameters accounting for the system resolution such as the noises from the components, e.g. light source/detector, or environmental fluctuations. Conventional SPR sensors are still considered the “gold standard” in refractometric sensing of molecular binding interactions over large areas: They exhibit very high bulk RI sensitivity (up to ~10,000 nm/refractive index unit (RIU)), minimum detectable RI changes (resolution) of 10^−7^ RIU, and a limit of detection of a few picograms of protein/cm^2^^3^. Nanoplasmonic sensors generally exhibit much lower bulk sensitivity, but can still show comparable thin-film sensitivity due to their tighter confinement of plasmon fields^1^. Sophisticated nanoplasmonic resonators can also exhibit sharper resonance linewidths (hence improved FOM and larger intensity change at a given wavelength) than conventional SPR. However, if sharp resonances are obtained at the expense of reduced photon counts or higher system noise, the overall detection limit may be degraded^4^.

## New generation of nanoplasmonic sensors

**Fig. 1:**
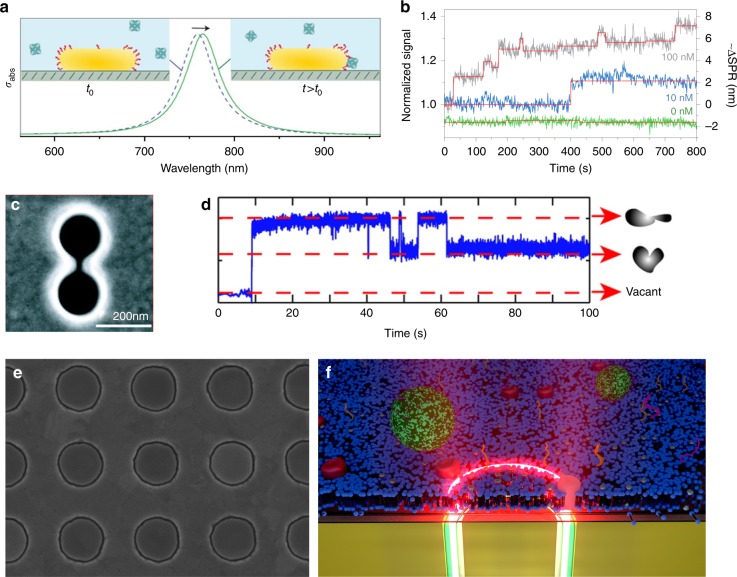
Nanoparticle, tip, hole, and gap for sensing and manipulating biomolecules. **a** Gold nanorod for label-free plasmonic detection of a single nonabsorbing protein molecule. Reprinted from ref. ^5^ with permission. Copyright 2012 Springer Nature. **b** Time-resolved photothermal signal for biotin-functionalized gold nanorods in the presence of protein (streptavidin-R-phycoerythrin conjugate) molecules. Reprinted from ref. ^5^ with permission. Copyright 2012 Springer Nature. **c** A double-nanohole aperture was used for low-power optical trapping and label-free detection of a bovine serum albumin molecule. Reprinted from ref. ^8^ with permission. Copyright 2012 American Chemical Society. **d** A time trace of the optical power transmitted through a double-nanohole in a gold film, using a solution containing protein bovine serum albumin (BSA) molecules. Reprinted from ref. ^8^ with permission. Copyright 2012 American Chemical Society. **e** High-throughput atomic layer lithography^9^ technique allows large-scale fabrication of coaxial apertures with sub-10-nm gaps, which can be used for surface-enhanced spectroscopies and optical trapping. Scanning electron micrograph of an array of gold coaxial nanoapertures (250 nm diameter and 10 nm gap width). Image credit: Daehan Yoo. **f** By interfacing nanoplasmonic structures with fluid biomembranes, it is possible to study dynamic interactions of lipids, proteins, and nanovesicles. This illustration shows a proposed scheme of optically trapping a single membrane protein molecule in a supported lipid bilayer membrane. Image credit: Christopher T. Ertsgaard

While conventional SPR sensors excel at measuring molecular binding interactions over large ensembles and rather macroscopic areas, nanoplasmonic structures can perform other unique tasks such as single-molecule detection, optical trapping, and rapid pre-concentration, thus creating their own new set of performance metrics. By pushing the plasmonic field confinement in all three dimensions via a gold nanorod, researchers have accomplished label-free detection of a single protein molecule (Fig. 1a, b)^5^. Nanoplasmonic structures can also boost gradient optical forces^6,7^. Pushing the limit of this principle, a double-nanohole was used for low-power optical trapping of a single protein (Fig. 1c, d)^8^. With large-scale fabrication of single-molecule trap arrays based on coaxial nanoapertures (Fig. 1e)^9^, next-generation sensors could enable massively parallel trapping and conformation dynamics sensing of single molecules without tethers/fluorophores. One potential limitation of surface-based trapping is an immobilization of the molecule upon trapping. One could overcome this challenge by covering nano-optical traps with fluid biomembranes (Fig. 1f), which will open up possibilities to trap single membrane proteins and study their interactions with lipid rafts and nanovesicles.

Even with their remarkable sensitivity and strong near-field trapping capabilities, plasmonic sensors can suffer from the fundamental limitations of diffusion. For sensing low-concentration analytes, their diffusion into the nanometric sensing ‘hotspot’ can lead to long waiting times. Fortunately, many nanoplasmonic structures (sharp tips, holes, nanogaps) can be designed to generate long-range forces to speed up the delivery of analytes to hotspots. Researchers have added innovative schemes onto nanoplasmonic sensors to accelerate mass transport, including electrokinetic pre-concentration or flow-through sensing^1^. With on-chip integration of these concentration schemes, nanoplasmonic sensors may be able to surpass conventional SPR when key performance metrics—limit-of-detection, response time, and minimum sample volume—are considered simultaneously.

## Surface chemistry and biological interfacing

While the sensitivity, resolution, and detection speed are important metrics to evaluate the nanoplasmonic “hardware”, another critical element is the surface chemistry to interface the hardware with ‘soft’ biological matter. Regardless of how well the underlying sensor performs, the outcome of biosensing is determined via molecular recognitions between receptors and targets. Nanoplasmonic sensors often exhibit nonplanar and heterogeneous surfaces, presenting challenges for surface modification strategies. This issue can be addressed by adding a thin conformal silica shell to enable well-established silane chemistry and facilitate biomembrane formation^10,11^. Interfacing nanoplasmonic sensors with biomembranes can also allow the incorporation of membrane-bound receptors and passivate the surfaces against nonspecific binding^4^.

Microarray technologies for nucleic acids and proteins have been revolutionizing screening efforts in many ways, and we consider this application one of the more promising avenues for integration with nanoplasmonic sensors toward parallel and label-free measurements of molecular binding kinetics^1^.

## Nanoplasmonics for infrared spectroscopy

Infrared (IR) absorption spectroscopy probes vibrational modes associated with the molecular bonds of a sample and provides nearly unparalleled chemical information. Despite this unique advantage, poor sensitivity and difficulties in sampling in aqueous solutions limit the application of infrared spectroscopy for measuring biological analytes in real time. The limited sensitivity is due to inefficient coupling of long-wavelength mid-IR radiation to the small absorption cross-section of molecular vibrations. This poses a significant challenge to probe thin layers of biomembranes, protein monolayers, and low-concentration analytes. For measurement in aqueous solutions, the strong O–H absorption bands of water molecules can overwhelm the signal from biological samples.

While SPR can measure receptor-ligand binding kinetics, it cannot identify bound analyte molecules. As one of the promising options to perform such molecular “fingerprinting”, we see surface-enhanced infrared absorption spectroscopy (SEIRA) as an exciting opportunity with many open challenges. Nanoplasmonic structures can provide well-defined resonances with strong near-field enhancements and sub-wavelength light confinement in a reproducible manner. By overlapping these resonances with the absorption bands of targeted molecules, specific molecular bonds can be selectively excited and large signals from ultra-small sample volumes can be achieved. Researchers have been adapting SEIRA for ultrasensitive detection of various thin-film specimen^12–15^. In addition, the strong light confinement of plasmonics brings advantages for in-situ measurements. The field intensity decays exponentially from the plasmonic substrate surface with a penetration length of <100 nm. Thus, one can selectively probe surface-adsorbed molecules while minimizing the interference from the rest of the sample (i.e., water molecules in solution). Interestingly, the field enhancement for SEIRA extends sufficiently away from the surface (~100 nm)^16^ in comparison to SERS whose enhancement is limited to only a few nanometers. This feature makes SEIRA ideal for studying proteins, biomembranes, as well as nanovesicle cargo while monitoring their dynamic interactions in real-time.

## Detecting conformational changes

**Fig. 2:**
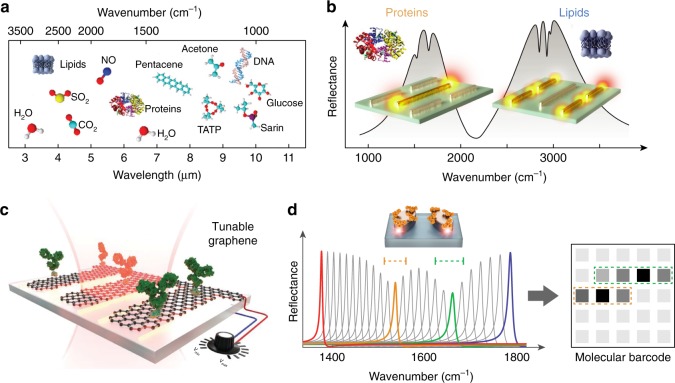
New directions in surface-enhanced infrared spectroscopy. **a** The mid-infrared spectral range contains characteristic absorption signatures associated with the vibrational modes of a wide range of molecules and therefore provides rich chemical information. Adapted from ref. ^13^ with permission. Copyright 2017 American Chemical Society. **b** Plasmonic nanoantennas can focus IR light into nanoscale volumes to enhance and detect the absorption fingerprints of small amounts of molecules on the surface. Multi-resonant antennas simultaneously monitor lipid membranes and protein molecules to unravel their interaction kinetics. Adapted from ref. ^17^ with permission. **c** In contrast to static metallic antennas, novel materials such as graphene enable dynamically tunable resonances via external electrostatic biasing. Reprinted from ref. ^18^ with permission. Copyright 2015, American Association for the Advancement of Science. **d** High-Q dielectric metapixels can overcome intrinsic metal losses to convert absorption signatures into barcode-like molecular images without the need for spectrometry. Adapted from ref. ^20^ with permission. Copyright 2018, American Association for the Advancement of Science

Notably, the amide-bond signatures of proteins resulting from backbone vibrations can provide comprehensive insights on the protein structure and conformation. Detection of protein conformations is crucial to understand the underlying role of protein misfolding in incurable neurodegenerative disorders including Alzheimer’s and Parkinson’s diseases. Critically, these conformational changes do not involve mass transfer and thus are largely inaccessible to other methods. The potentials of plasmonics to access rich mid-IR spectral content for functional protein studies are yet to be exploited. Specifically, we think that a critical scientific challenge on this front is to show real-time dynamic analysis of secondary structure changes in native proteins with single-molecule resolution. Another challenge is to analyze proteins and their conformations in heterogeneous biological samples. Differentiating and monitoring selected biomolecules in complex mixtures containing diverse analytes such as lipids, nucleic acids, and small molecules is a central goal in biosensing. Infrared spectroscopy, by providing exquisite chemical selectivity, is uniquely positioned for such tasks. A multi-analyte biosensor could be achieved with substrates supporting multiple resonances that are designed to match the characteristic vibrations of different analytes of interest. In this regard metasurfaces and metamaterials can provide new ways to engineer unconventional propagating and resonant modes. Recently, a multi-resonant metasurface enhancing simultaneously amide and methylene bands has been used to resolve the interactions of lipid membranes with polypeptides and peptide-induced neurotransmitter cargo release from synaptic vesicle mimics (Fig. 2b) in real-time^17^.

Another avenue to extend the functionality of plasmonic sensors is through dynamic tunability. Noble metals, in particular gold, have been the material choice for SEIRA due to their strong plasmonic resonances and biocompatibility. The resonances of metallic structures, however, are fixed upon fabrication. In contrast, two-dimensional (2D) materials such as graphene are emerging as a new platform to provide dynamically tunable resonances and reconfigurable biosensors via electrostatic biasing (Fig. 2c)^18^. Furthermore, graphene can simultaneously function as electronic sensors and even atomically sharp tweezers to trap molecules on its edges^19^. On the other hand, these materials come with their own limitations. Graphene supports weak plasmonic resonances because of its low oscillator strengths. The resonances of hexagonal boron nitride (hBN) are spectrally limited in device applications due to the fixed phonon bands. Hybrid substrates containing multiple materials such as graphene, hBN, or metallic antennas can be a viable solution to better exploit tunability and optical conductivity. Likewise, complementary materials such as low-loss dielectrics supporting naturally high-*Q* resonances are promising for SEIRA. For example, a recent work employs arrays of high-*Q* dielectric metasurface pixels to convert near-field-enhanced absorption signatures into 2D molecular barcode-like images (Fig. 2d)^20^.

Compact on-chip integration of mid-IR sensors has been challenging partly due to the limited choice of efficient light sources and sensitive detectors. Fortunately, the field is advancing rapidly with the introduction of widely tunable room-temperature quantum cascade lasers, optical parametric oscillator sources, on-chip frequency combs, broadband fiber sources, large-area imaging detectors, and maturing mid-IR waveguide technology. These advancements will bring new opportunities by eliminating the need for expensive and bulky Fourier Transform IR spectrometers and moving mechanical parts.

As innovations by many researchers helped overcome the challenges of manufacturing nanoplasmonic structures, we expect that the next round of exciting developments will move towards compact systems integration, ultrasensitive spectroscopies of complex samples, and label-free measurements of single-molecule dynamics, which could advance fields as diverse as point-of-care diagnostics, drug discovery, structural biology, and environmental monitoring.
